# Controlling T cells spreading, mechanics and activation by micropatterning

**DOI:** 10.1038/s41598-021-86133-1

**Published:** 2021-03-24

**Authors:** Anaïs Sadoun, Martine Biarnes-Pelicot, Laura Ghesquiere-Dierickx, Ambroise Wu, Olivier Théodoly, Laurent Limozin, Yannick Hamon, Pierre-Henri Puech

**Affiliations:** 1 Adhesion and Inflammation Lab (LAI), Aix Marseille University, LAI UM 61, 13288 Marseille, France; 2 Adhesion and Inflammation Lab (LAI), Inserm, UMR_S 1067, 13288 Marseille, France; 3 Adhesion and Inflammation Lab (LAI), CNRS, UMR 7333, 13288 Marseille, France; 4 Centre d’Immunologie de Marseille Luminy (CIML), Aix-Marseille University, CNRS, Inserm, CIML Marseille, 13288 Marseille, France; 5 Present Address: Division of Medical Physics in Radiation Oncology, German Cancer Research Center (DKFZ), Heidelberg, Germany; 6 Present Address: Department of Biophysics, University of Wrocław, Wrocław, Poland

**Keywords:** Biophysics, Membrane biophysics, Cell biology, Cell adhesion, Immunology, Adaptive immunity, Lymphocytes

## Abstract

We designed a strategy, based on a careful examination of the activation capabilities of proteins and antibodies used as substrates for adhering T cells, coupled to protein microstamping to control at the same time the position, shape, spreading, mechanics and activation state of T cells. Once adhered on patterns, we examined the capacities of T cells to be activated with soluble anti CD3, in comparison to T cells adhered to a continuously decorated substrate with the same density of ligands. We show that, in our hand, adhering onto an anti CD45 antibody decorated surface was not affecting T cell calcium fluxes, even adhered on variable size micro-patterns. Aside, we analyzed the T cell mechanics, when spread on pattern or not, using Atomic Force Microscopy indentation. By expressing MEGF10 as a non immune adhesion receptor in T cells we measured the very same spreading area on PLL substrates and Young modulus than non modified cells, immobilized on anti CD45 antibodies, while retaining similar activation capabilities using soluble anti CD3 antibodies or through model APC contacts. We propose that our system is a way to test activation or anergy of T cells with defined adhesion and mechanical characteristics, and may allow to dissect fine details of these mechanisms since it allows to observe homogenized populations in standardized T cell activation assays.

## Introduction

The engagement by the T lymphocyte receptor αβ (TCR, T cell receptor) of molecules of the major histocompatibility complex loaded with agonist peptide (pMHC) is a central step in the adaptive immune response^1^. This recognition is characterized by its high specificity and sensitivity^2^, with a broad spectrum of intracellular signaling events leading to T cell activation and effector functions^3^. How T cells contrasting cellular responses like clonal expansion, anergy or apoptosis reflect the quantitative and qualitative diversity of antigens remains largely an enigma from a molecular and kinetic point of view^4–7^.

However, the main protein protagonists have been identified and extensively documented on both the antigen presenting cell (APC) and the T cell. At a minimum^8^, TCRαβ is an octameric polypeptide complex, composed of a clonotypic and hypervariable recognition module (chains α, β) and a transduction module formed by the CD3 invariant chains associated in dimers^9^. The binding of TCR to pMHC triggers the phosphorylation of ITAM units, located in the cytoplasmic domains of the CD3 subunits, by the kinase of the Src Lck family. This results in the recruitment and activation of kinases from the ZAP-70 Syk family, which in turn phosphorylate various critical signaling intermediates, including the LAT adapter^9,10^. A major gap in this picture is the lack of understanding of the mechanism by which the engagement of the TCR/CD3 ligand on the T cell surface causes the phosphorylation of CD3 chains by the Lck kinase located on the inner sheet of the plasma membrane, a process that is commonly referred as “TCR triggering”. Various models have been proposed so far to explain this^11^, including the molecular aggregation, pseudo-dimer, kinetic segregation or conformational change models^2,12,13^.

While all these different models are not fully consistent with each other, some of them may represent different facets of the same mechanism that are simply shifted in time or space. Finally, all these models attempt to explain the profound paradox that exists between, on one hand, the selectivity/sensitivity/rapidity of responses contrasting, on the other hand, with the diversity/scarcity of antigens exhibiting low affinities (as typically obtained by “3D vs. 2D” surface plasmon resonance) for TCR^4,14^.

Seminal studies^15–17^ measuring the kinetic parameters of 2D TCR/pMHC interactions (in a membrane environment) radically contradict studies conducted with molecules in solution and demonstrate very rapid association/dissociation cycles of the same TCR for the same pMHC, the affinity of TCR for its natural ligand being of the same order as the one between adhesive molecules such as ICAM1 and LFA1^18^.

All these parameters classify TCR at the heart of a paradigm of unconventional interactions between membrane receptors/ligands. Ultimately, the transverse organization of the plasma membrane (its composition, its inhomogeneities, the nature of its interactions) would influence the ability of T lymphocytes to respond to the antigen^19^.

Recently, and driven by technological, biophysical and biological developments, it has been proposed that forces at the interface of the T cell and APC while the former scans the latter may play a crucial role for T cell recognition and subsequent activation^18–21^. Experiments at single molecule scale have tested if TCR/pMHC bond rupture forces^22^ or lifetimes^23,24^ depends on the quality of the peptide and have proposed that this bond could exhibit a behaviour more complex than expected, namely a catch bond behaviour^23^, where, depending on the peptide, the lifetime of the bond can be modulated and even extended for a range of forces compatible with the ones that cell protrusions may exert^25,26^. The geometry of the force application has also been investigated using refined micromanipulation methods^27–29^. Moreover, the way the forces are exerted, continuously vs. intermittently, have been proposed to be an important modulator of recognition^23,30^, directly involving the T cell cytoskeleton in the recognition^31^. Aside, at single cell level, T cells have been observed to react differently as a function of the mechanics of the substrate they are interacting with^32,33^, in line with the recent demonstration that APCs exhibit different mechanical properties, which are modulated along the inflammation process^34^. The complexity of the response to substrate mechanics, modulated by the molecules used to make the cells spread on it, have been very recently examined^35^. Aside from cell mechanics, cell shape has been shown to have a non negligible impact on cellular functions^36^ and T cell activation^37^. Applied forces can also, in turn, affect the shape of the T cell itself^38^ and APC cytoskeleton may impact T cell recognition^39–41^.

Single cell fluorescence studies^42^ or force based measurements where cells are adhered on a substrate^22,30,31,37,43^ or using single T cell/APC interaction^44–46^, while highly informative, have the inherent drawback of relying on the parallel or more often sequential measurement of cells in a heterogeneous population, which may possess slightly different mechanics and shape. In this context, we recently proposed a technique to couple force based measurements using atomic force microscopy and optical tracking of calcium fluxes or opto-genetics perturbation of cell mechanics as a new tool^43^.

Here, we designed a strategy to “standardize T cells”, based on a careful examination of the activation capabilities of proteins and antibodies used for adhering T cells on micro-stamped substrates^47^. We applied it on a T cell line recapitulating activation steps up to Interleukin 2 production, independently of CD28 co-stimulation^42^. It allowed us to control at the same time the position, adhesion, shape, mechanics (as measured using atomic force microscopy indentation) and activation state of T cells (as followed by calcium fluxes). We examined the capacities of activation, using a soluble anti CD3 antibody, in comparison to T cells adhered to a continuously decorated substrate with the same density of ligands, and verified that T cells have a similar behavior in regard to classical activation assays. We further show that, with our system, CD45 mobilization on continuous substrates or patterns of controlled areas is not affecting the proper activation as observed through calcium fluxes. To confirm this, we expressed an exogenous adhesion molecule in T cells that can be used for immobilization purpose and demonstrated that this did not perturb T cell calcium response similarly as our anti CD45 strategy, hence suggesting this new construct as a new tool to dissect T cell activation for biophysical techniques which require adherent cells.

## Material and methods

### Chemicals

Chemicals and proteins were obtained from Sigma Aldrich except when noted below. Culture media and supplements were obtained from Gibco. References can be found in^42,43^ for generic components.

### Cell culture

#### Culture

3A9m T cells were obtained from D. Vignali^48^ and cultured in RPMI completed with 5% Foetal Bovine Serum (FBS), 10 mM Hepes in 5% CO2 atmosphere. COS-7 APC were generated as previously described^42^ by stably co-expressing the α and the β chains of the mouse MHC class II I-Ak, cultured in DMEM (5% FBS, 1 mM Sodium Pyruvate, 10 mM Hepes, and geneticin 10 µg/ml). Cells were trypsinized up to three times a week by treating them with either Trypsin/EDTA or 0.53 mM EDTA at 37 °C for up to 5 min.

#### Cell fixation and labelling for confocal imaging

3A9m cells were fixed using 4% Paraformaldehyde (20 min on ice), saturated with glycine 0.1 M and permeabilized with 0.1% Triton and labelled in presence or not of 0.01 mM Hoechst (at room temperature, 10 min) with 0.5 µM phalloidin-Alexa Fluor 488 (Life technologies) (at room temperature, 20 min).

#### Protein expression

The expression levels for TCR and CD45 (for T cell hybridomas), and MHCII (for COS-7 APC) were routinely monitored by flow cytometry (BD Biosciences, LSR2) once a week. Expression of CD4 was tested once for every cell batch since it was observed to be stable over time (not shown).

### Antibody production and labelling

#### Production

The antibodies used for this study were produced from the hybridoma collection of Centre d’Immunologie de Marseille-Luminy (CIML) (namely anti CD3 2C11, anti CD4 GK1.5, anti CD45 H193.16.3). Briefly, hybridoma were routinely grown in a complete culture medium (DMEM, 10% FBS, 1 mM sodium pyruvate) prior to switching to the expansion and production phase. They were cultured in DMEM with decreasing concentrations of low immunoglobulin FBS (Life Technologies) down to 0.5%. Cells were then maintained in culture for 5 additional days enabling immunoglobulin secretion prior to supernatant collection and antibody purification according to standard procedures.

#### Fluorescent labelling, when needed

200 µg of antibody solutions (in PBS 1X) were mixed with N-Hydroxysuccinimide (NHS) ester functionalized dye (dye/protein ratio set to 3:1) with sodium bicarbonate solution 0.1 M (pH 8.3). We used Alexa Fluor 488 and 647 (Thermofisher Scientific) or Atto 565 (Sigma Aldrich). After incubation for 1 h at RT under constant agitation, uncoupled dyes were separated from labeled antibodies by size exclusion chromatography (on Sephadex G25 PD10 columns GE Healthcare) followed by dialysis against PBS 1 × overnight at 4 °C. At last, a dialysis against PBS 1 × was performed for 1 hr. The concentration and labeling efficiency was then assessed on a Nanodrop 100 (Thermo Scientific). The obtained dye/protein ratio was typically between 1 and 2.

### MEGF10 stably expressing T cells

The MEGF10::EYFP construct has been previously described in^49^. Briefly the 220 last bases of the 5′ UTR together with the human cDNA coding for MEGF10 were subcloned upstream and in frame with the EYFP sequence into the pEYFP-N1 vector. The plasmid was introduced into 3A9m T cells by AMAXA nucleofection (Kit V, B024; Lonza) according to the manufacturer and as already detailed in ref.^50^.

### Continuous substrates

#### LabTek chambers for calcium imaging

8 well-LabTek II chambers (Nunc), with glass bottom, were coated by adsorption of solutions of antibodies in PBS 1 × overnight at 4 °C, then saturated with PBS 1 ×, 1% BSA solution, for 1 h at 37 °C prior to observations.

#### Glass bottom Petri dishes

##### Antibodies coating

Glass bottom Petri dishes (WPI Instruments, Fluorodish FD35-100) were incubated with 50 µL of anti CD45 H193.16.3 antibodies (final 50 µg/ml) for 45 min at room temperature. Once the droplet was removed, the surfaces were extensively rinsed first with sterile PBS 1X, then with HBSS-H.

##### Poly-l Lysine coating

The petri dishes were activated with residual air plasma for 10 min, then incubated with a mix 9:1 poly-L-lysine:Alexa Fluor 546-labelled poly-l-lysine solution (0.1% or 0.01% w/v H_2_O, Sigma P8920-100 ml, labelled with Alexa Fluor 546 NHS Ester Life Technologies A20102 following provider protocols) for 45 min at room temperature, and rinsed as above.

### Microstamped substrates

#### Stamps production

Stamps were produced using soft photolithography techniques from in-house designed patterns. They consist of regularly spaced disks of size between 5 and 50 µm in diameter. The spacing was optimized so that one T cell could not adhere to more than one pattern. For the smaller sizes, immobilization was not very efficient for us (e.g. 5 µm), while for larger ones (e.g. 50 µm), several cells may adhere to the same pattern (not shown). The diameters used here were 10, 15 and 20 µm.

To produce the stamps, we used PDMS (Sylgard 184, Dow Corning) in a ratio w/w 1:10 of curing agent. 1 cm^2^ stamps of typically 0.5 cm thickness were then cut using a scalpel. The stamps were then washed in two steps in a sonicator at maximal power: (i) 20 min at 65 °C with 5% Decon; (ii) 20 min at 65 °C in ultrapure water. Finally, they were dried under gentle nitrogen flux and stored protected from dust before immediate use.

#### Stamping procedure

50µL of the desired stamping solution (typically 10–100 µg/ml of protein) was spread onto the stamp and let adsorb during 45 min at room temperature, protected from light. The droplet was then aspirated and the stamp briefly dried using a nitrogen flux, at room temperature. The stamp was immediately gently pressed using tweezers on a clean glass bottom Petri dish and the transfer was let to occur for 10 min in a cell culture incubator at 37 °C, protected from light. After removing the stamp with great care, the substrate was intensively rinsed with 0.2 µm filtered PBS 1 × before adsorption of the desired complementing molecule (typically 10–100 µg/ml of protein, with an optimal e.g. for anti CD45 antibody at 50 µg/ml when the first one was (b) BSA, see text) for 1 h at room temperature, protected from light.

Final extensive rinsing was performed with the desired experimental buffer (here, HBSS/1 mM Hepes) and the final volume in the Petri dish was set to 2–3 mL for further experiments. Whenever possible, the quality of the stamping was checked by fluorescence either of the stamped molecule or of the complementary one, or after adding e.g. fluorescent streptavidin over biotinylated substrates.

#### Cell adhesion on stamps

Cells were seeded over the stamped region and let to adhere and spread for 1 h in controlled conditions, protected from light. After observations in transmission, fluorescence and eventually interference microscopy imaging (RICM, see below), the non adherent cells were removed by a gentle rinsing step with HBSS and the remaining adhered cells were let to rest for at least 15 min before starting any experiment.

### Calcium imaging

The measurements of 3A9m cytosolic calcium fluxes were performed according to the “methods for automated and accurate analysis of cell signals” (MAAACS) extensively described in Ref.^42,50^.

#### Calcium reporter loading

In brief, 3A9m cells were loaded with PBX calcium reporter (BD) diluted in 1X dye loading solution at 37 °C for 1 h in the dark, according to manufacturer’s instructions. Cells were then washed twice by gentle centrifugation and resuspended in Hank's balanced salt solution buffered with Hepes (1 mM) (HBSS-H). They were then introduced in the desired observation chamber (Labtek or glass bottom Petri dish).

#### Confocal microscopy

Analysis was performed using a Zeiss LSM 780 confocal microscope equipped with a C-Apochromat 40 ×/1.2 water immersion objective as well as an argon laser with a 488 nm dichroic and a 505–530 nm band pass filter. A temperature control system was used to ensure that 37 °C was maintained during the entire acquisition. Time-lapse movies were typically made of 150 images taken every 7 s (with a pinhole set to 4 airy units), while cells were kept at 37 °C using a hot plate. When needed, a similar second series of acquisitions was launched upon activation with soluble anti CD3 antibodies (2C11; 20 µg/ml) or a suspension of COS APC cells loaded or not with the desired peptide (1 million cells/ml).

#### Quantification

Calcium response intensity parameters as well as cell displacement (as “speed”) and cell shape (as “circularity”) were determined by MAAACS as previously described^42^. Specific calcium response amplitude was determined upon specific thresholds (depending on experimental stimuli) set according to our previous studies. The percentage of responding cells, average response amplitude and average response time fraction were automatically calculated and tabulated in MS Excel data sheets. Results were plotted as dot plots, limited to calcium responses above threshold.

### Reflection interference contrast microscopy (RICM)

A Zeiss Axiovert, equipped for fluorescence microscopy (GFP, TRITC filter sets) and a specific Antiflex 63 × objective (Zeiss) was used to monitor the contact zones of cells with substrates^51–53^. The RICM was performed at 546 nm wavelength by using a dedicated filter set. Dark zones in the images were the signature of close apposition of the membrane, to the few nanometers scale, to the substrate and quantified using either hand selection or homemade macros in Fiji/ImageJ software^54^. From the ROI we obtained, adhesion area and adhesion perimeter were measured, and we calculated a shape index for each as 4π × Area/Perimeter^2^. The closer to 1 the more circular the shape of the contact zone was; at 0.5, the shape was elongated.

### AFM set-up

The set-up has been described in details elsewhere^43^. Measurements were conducted with an AFM (Nanowizard I, JPK Instruments, Berlin) mounted on an inverted microscope (Zeiss Axiovert 200). The AFM head is equipped with a 15 μm z-range linearised piezoelectric scanner and an infrared laser. The set-up sits on an active damping table (Halcyonics). AFM was used in closed loop, constant height feedback mode. Temperature control was achieved using a Petri Dish Heater module (JPK Instruments), which controller was connected to the AFM one.

Bruker MLCT-UC cantilevers were used in this study, and glass beads (5 µm in diameter, silica beads from Kisker Biotech GmbH, larger than cantilever tip) were glued at their extremity using micropipette micromanipulation with UV optical glue (OP-29, Dymax) and intense UV curing [10 min at maximal power of a UV oven (BioForce Nanosciences)]. The sensitivity of the optical lever system was calibrated on the glass substrate and the cantilever spring constant by using the thermal noise method^55^, using JPK SPM software routines (JPK Instruments) in situ at the start of each experiment. The calibration procedure for each cantilever was repeated three times to rule out possible errors and spring constants were found to be consistently close to the manufacturer’s nominal values; the calibration was stable over the experiment duration.

The inverted microscope was equipped with 10x, 20xNA0.8 and 40xNA0.75 lenses and a CoolSnap HQ2 camera (Photometrics). Bright field images were used to select cells and monitor their morphology during force measurements. For fluorescence, the microscope was equipped with a LED illumination system (Colibri 2, Zeiss) and suitable filter sets. Images were obtained through either Zen software (Zeiss) or µManager^56^.

### T cell mechanics using AFM

In order to measure the Young modulus of the shaped and non shaped cells, AFM indentation experiments were performed using MLCT-UC levers on which a bead, smaller than the cell, was glued (see above). Cells were adhered at 25 °C when loaded with calcium reporter dye to avoid compartmentalization and 37 °C overwise^50^ and were selected by bright field examination and, if needed, the protein printed pattern was examined using fluorescence microscopy. The indentation experiments were then performed at a prescribed, constant, temperature (25 or 37 °C). The occurrence of dye compartmentalization^57^ limits the duration of the experiment to 1h30.

To start, the bead was positioned as much as possible above the center of the cell. The maximal force to be applied was set at 500pN (leading to indentation depths of the order of one µm, smaller than the typical cell size), the contact duration from zero (pure elastic deformation, the most common experiments) to eventually up to 10 s (to follow the viscous relaxation), the speed of pressing and pulling at 2 µm/s in all cases for a 7 µm displacement. Depending on the measurement, a single or up to 10 force curves were recorded for each cell, with a delay time of 1 s for repeated acquisitions in order to let the cell recover. Data was recorded at 2048 Hz.

For determination of the Young modulus, each force curve was examined by eye and processed with the “Hertz model procedure” included in JPK DP software (JPK Instruments)^58^: corrections for baseline, possible tilt of the baseline and Hertz model for spherical tips were applied, making the hypothesis that the cell behaves as an uncompressible material^59^. In short, the Hertz model allows to extract the elasticity of a material, as measured as its Young modulus, from the relation between the force, applied here by a spherical indenter onto the material, and the indentation, i.e. the corresponding penetration depth of the indenter. The higher the Young modulus is, the stiffer the material is. Here, only a subset of the entire force span (from the baseline to the maximal contact force) was fitted : we chose to fit over 0.5 µm of indentation to minimize contributions from the nucleus (see “Results and discussion”). Young modulus were found to be coherent with published ones for T and immune cells^34^.

For the evaluation of the force relaxation, we estimated a power-law exponent *n* for *f(t)* as *f* = *f*_*0*_*(t/t*_*0*_*)*^*n*^ following^60^. We verified that, when maintaining the piezo position constant, the relaxation induced only a moderate (~ 4–5% in average) variation of the cell's indentation. Then, we applied an ad hoc fit (using a dedicated Python procedure) and extracted *n.*

For each parameter, a median value per cell was then calculated and tabulated in each condition. The data was then plotted using Python libraries Matplotlib (matplotlib.org/) and/or Seaborn (stanford.edu/~mwaskom/software/seaborn/). We validated this way of pooling the data experimentally since no obvious correlation between the Young modulus and the force curve number (corresponding to the « mechanical history » of the cell) was observed (not shown).

### Data processing and statistics

Data was presented as data points with median or mean ± SEM otherwise precised. Data plotting and significance testing were performed on Linux or Windows PC 64 bits machines using Python (packages Seaborn, Matplotlib, Scipy, Numpy, Scikit) and/or R/Rstudio (http://cran.rstudio.com/, packages asbio and pgirmess for Kruskal–Wallis or Wilcoxon tests) and/or Graphpad Prism (6 or 7)^67^.

## Results and discussion

The build-up of a system where T cell localization, adhesion and shape, mechanical properties and activation level were controlled was divided into successive steps: (i) identifying relevant molecules to immobilize cells on a surface with cells keeping a low induced activation while remaining activable e.g. using soluble molecules (we refer further to this capacity as activability), (ii) determining a reproducible micropatterning protocole for shaping and controlling the spreading of small and reactive T cells in the frame of (i), (iii) characterizing their mechanical properties such as elasticity, (iv) comparing their activability to more classical conditions and (v) qualifying novel methods of immobilization using a new molecular construct that can be easily transfected into T cells.

### Optimizing T cell adhesion while controlling their activation level

T cells are mostly non-adherent cells in culture, so that the first step was to have them adhere on glass substrates, considering that the substrate should immobilize the T cells while keeping their activability. Adherent and repellent molecules were tested, in order to create afterwards adhesive patterns surrounded by non-adhesive areas, therefore controlling their overall shape and spreading.

**Figure 1:**
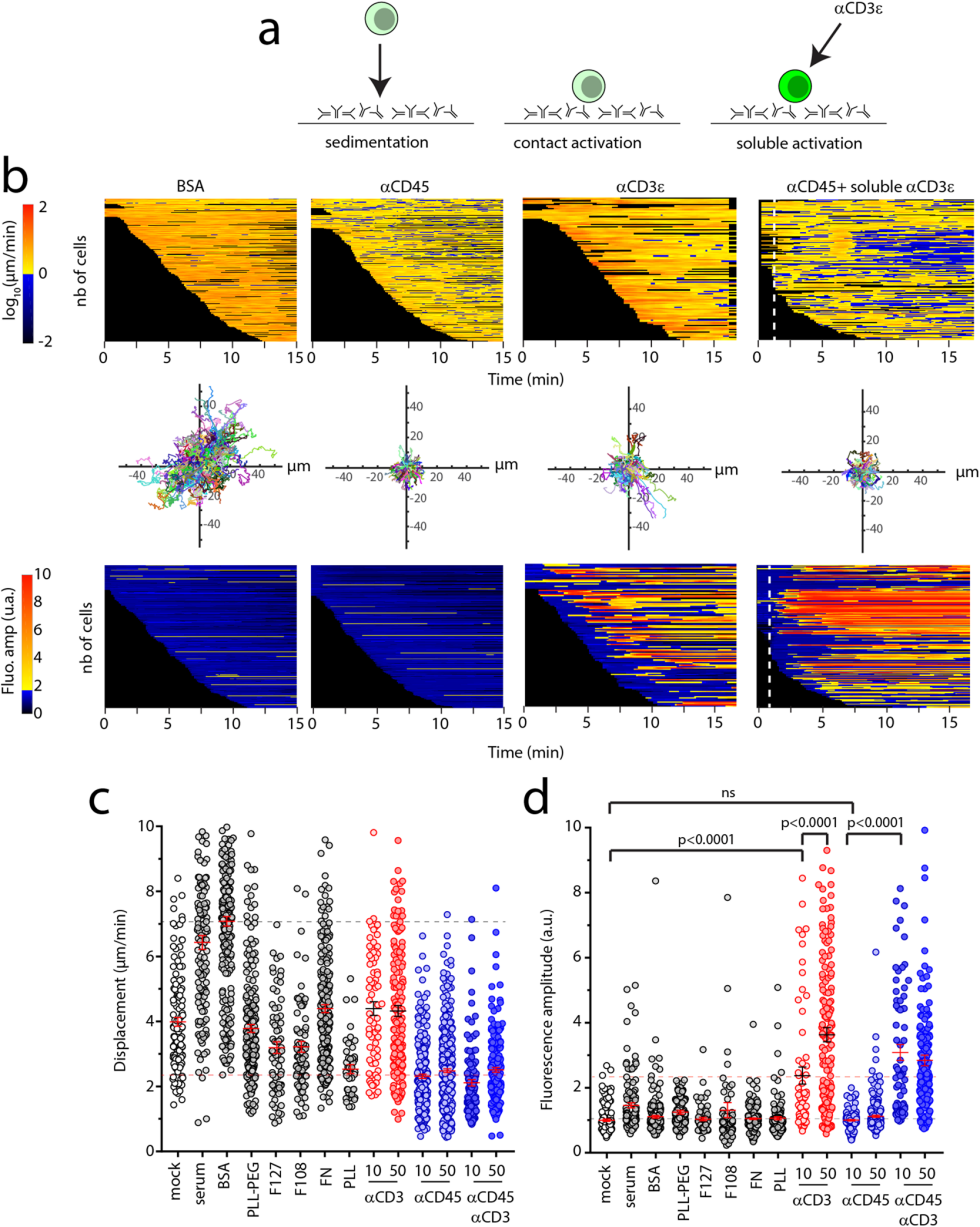
(**a**). Schematics of a MAAACS^42^ experiment, where cells first sediment on a substrate which may be passivated, adherent or activating and were, in some cases, further stimulated with soluble anti CD3 antibodies. (**b**) Heatmaps of cell displacement, re-aligned cellular trajectories (over the duration of the acquisition, typically 15 or 30 min) and heatmaps of normalized calcium fluxes vs. time extracted from representative movies on (i) a non adhesive substrate (BSA 1%), (ii) an adhesive but non activating substrate (anti CD45 antibody), (iii) an adhesive and activating substrate (anti CD3 antibody), and (iv) on cells after landing on (ii), but subjected to reactivation via soluble anti CD3 antibody (the instant of injection is presented as a dotted line). One line corresponds to one detected cell. (**c**) Measured displacement of cells, from MAAACS (see text), which is used to define the adhesiveness of the tested substrates: when average displacement is < 3 µm/min, the substrate is defined as adhesive. Dotted lines were shown for min and max mean values. (**d**) Fluorescence amplitude of cells, corresponding to substrates tested in **c**. Dotted lines were shown for min and anti CD3 mean values. Mock condition corresponds to non treated, bare glass. Data was presented as scatter plots (1 point per cell) with mean ± SEM. Significance of difference was assessed with Mann–Whitney test.

We tested a large number of putative adherent and repulsive coatings (Fig. 1a), with dose dependent conditions, at 37 °C, whose properties were evaluated using the MAAACS algorithm^42^, a refined cell tracking detection software which includes quantification of intracellular fluorescent signals such as calcium fluxes together with cell shape and displacement (Fig. 1b). Here, we took advantage of the velocity as measured by the algorithm as a convenient readout of the capacity of our panel of candidate molecules to immobilize cells (Fig. 1c). Aside, we evaluated whether those compounds were prone to activate or to affect T cell activability, by recording the calcium response of cells once they reached the surface, followed, when applicable, by activation with saturating concentration of soluble aCD3 (Fig. 1d).

We observed that T cells seeded onto surfaces coated with activating anti CD3 antibody (2C11 clone) were adhering (i.e. instant displacement drops rapidly after contact) and activating strongly (rise of the fluorescence amplitude of the PBX calcium indicator), while on glass surfaces decorated with an anti CD45 antibody (H193.16.3 clone), T cells were strongly adherent without inducing a rise of calcium influx (consistent with our previous observations^42^) unless subsequently stimulated with soluble anti CD3 antibody (Fig. 1d). To the contrary, T cells did not adhere on serum-, PLL-PEG-, F127-, F108-, (biotin)-BSA-coated surfaces. On PLL, we noted that cell adhesion appeared to be insufficient to prevent T cells from detaching upon soluble anti CD3 antibody stimulation. Biotinylated BSA + streptavidin coating induced for some T cells only a slight activation compared to BSA or biotinylated BSA alone on continuous substrates but this coating was essentially non adherent, hence classifying it as repulsive and non activatory. On all other tested conditions (namely fibronectin, mICAM1, WGA, concanavalin A, protein A [without anti CD3 or anti CD45], anti CD43 (Santa Cruz, clone sc-7055) or anti MHCI (Biolegend clone 36.7.5) antibodies, following Ref.^25^ (not shown here) T cells were either loosely adherent and/or activated by the substrate, showing calcium signal fluctuations over threshold (Fig. 1c,d).

Due to its abundance and its large extracellular domain, CD45 has been used by many authors over years as a tool molecule, which can be used to efficiently immobilize T cells without strongly perturbating them^61^, although some reports indicate that CD45 segregation may nevertheless impact T cell activation^62^, depending on the isotype of the antibody used^63^. Recent reports show that CD45 segregation is not required to stimulate Jurkat T cells by immobilized recombinant monovalent antibodies raised against CD3^64^.

In order to evaluate the extend of CD45 immobilization, we performed 3A9m T cells surface labeling (with AF488-H193.16.3) prior or after seeding labeled cells onto non fluorescent H193.16.3 or poly-L-Lysine (PLL) coated Labtek culture chambers. We calculate the percentage of the fluorescence intensity on contact with the contact surface. We obtained a low fluorescence ratio (in %) when cells were seeded prior labeling in a more pronounced way on PLL than on anti CD45 antibodies (1.42 ± 0.86 vs 3.72 ± 1.45) (Suppl. Fig. 1a,b). On the opposite configuration (labeling prior seeding) we calculated a low enrichment at the basal membrane when labeled cells were seeded onto anti CD45 coated surfaces (5.69 ± 1.98) but stronger when seeded onto PLL (12.33 ± 3.83) (Suppl. Fig. 1c).

This low enrichments might be correlated with the rapid exchange and the mild affinity of the anti CD45 for its preys, supporting cell spreading without triggering intracellular signaling. In addition, actin cortex architecture was found homogeneously structured with small actin foci, consistent with a quiescent state of the cells (not shown).

This therefore qualified the H193.16.3 anti CD45 antibody as a candidate which fulfills the majority of our initial requirements, even using high concentrations of coated antibody (up to 50 µg/ml overnight incubation).

### Adhesive/repulsive micropatterns for T cells shaping

We chose to pattern protein disks since (i) this shape offers the largest adhesive area possible, (ii) it allows for a simple geometry to test from Atomic Force Microscopy (AFM) indentation experiments, e.g. for placing the indenter on reproducible part of the cell, with a known nucleus position (see section *T cell mechanics of patterned T cells*), (iii) it is known that T cells clones tend to become more spherical upon activation^65^ and (iv) all of this will contribute in our effort to minimise data dispersion.

**Figure 2:**
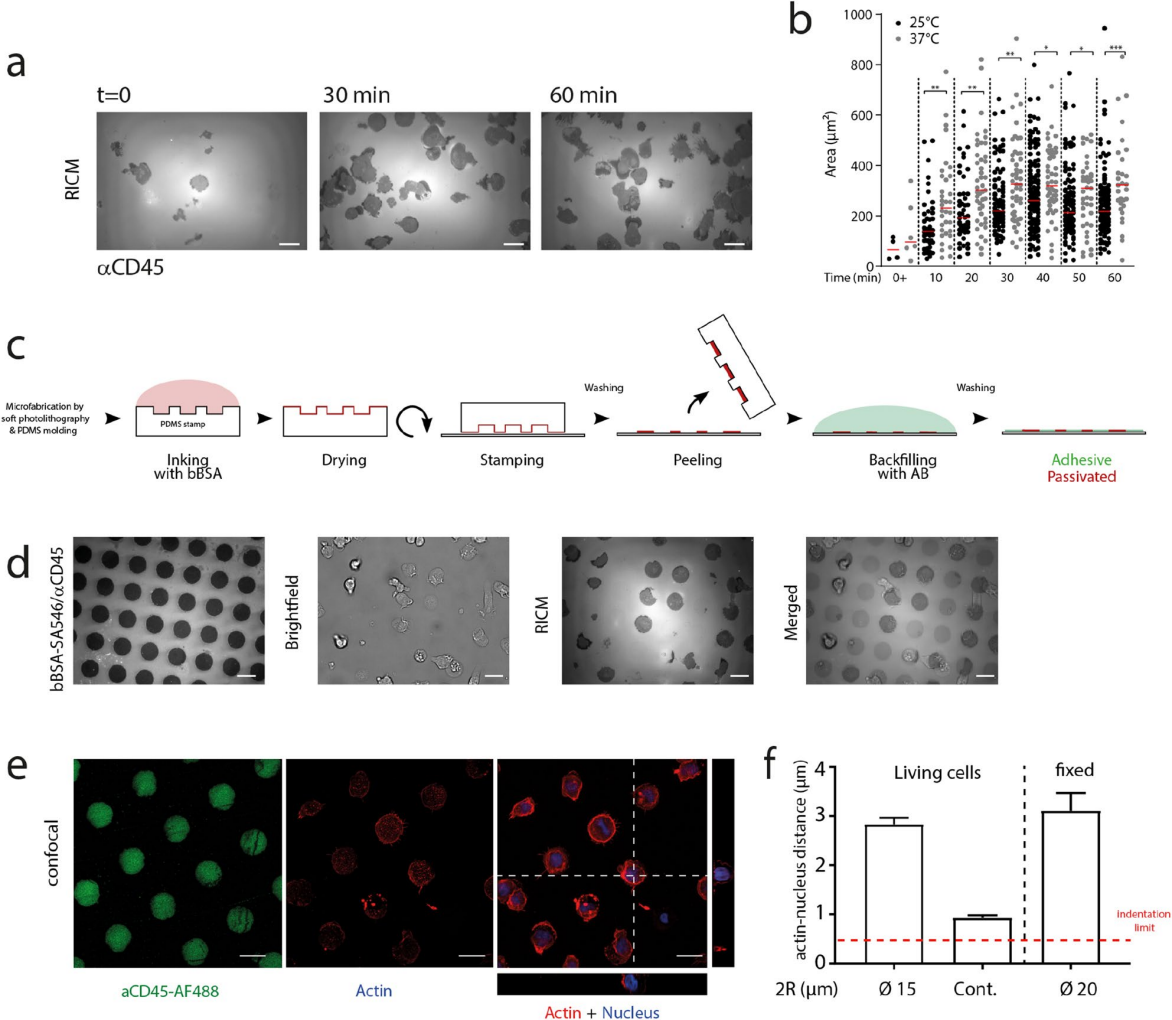
(**a**) RICM images of adhesion and spreading of 3A9m cells on anti CD45 “continuous” (here denominated as “cont.”) substrate at given time points. (**b**) Adhesion area on anti CD45 antibodies of cells vs. time at 25 °C and 37 °C. Each point represents a cell, and the bar denotes the median value. Statistical differences between temperatures at each time point were assessed using Mann–Whitney test. The adhesion after 60 min at 37 °C corresponds to an equivalent disk of diameter of ~ 20 µm. (**c**) Schematics of the inverse microstamping method, where the repulsive molecule (BSA or bBSA) is stamped first, before adsorbing the adhesive one (anti CD45) in the free zones. (**d**) Images of obtained substrates: (i) fluorescence image of the repulsive zones, completed with non labeled anti CD45 antibodies in the patterns (dark), (ii) brightfield images of 3A9m cells after 60 min incubation, (iii) corresponding RICM image of the adhesion zones, (iv) overlay of the three previous pictures. (**e**) Confocal imaging of 3A9m cells showing patterns of anti CD45 antibodies (green) cortical actin (red) and nucleus (blue), with projections of the z-stack. (**f**) Estimation of the distance between the cortical actin and the nucleus (mean+/SEM), to determine the maximal indentation for the Hertz-like fit (dotted line, see text). Scale bar = 20 µm.

To design our micropatterning strategy, we quantified the spreading area of 3A9m cells on continuously decorated aCD45 substrates vs. time over relevant periods of time (up to 1 h) by RICM (Fig. 2a,b). For these experiments, data dispersion arose from the dispersion of arrival time, ie. spreading history, of the cells in our fields of view, due to injection and sedimentation, which can be also appreciated in the MAAACS experiments (Fig. 1b). From the maximal area the 3A9m cells spread at 37 °C, we then estimated an equivalent maximal diameter of our circular patterns (2R ~ 20 µm), hence limiting our designs to smaller dimensions. In our hands, patterns smaller than 10 µm in diameter were not able to properly immobilize the T cells which would have been a major impairment for AFM experiments. As a consequence, the diameters which were used here were 10, 15 and 20 µm.

We first successfully directly stamped disks of Alexa Fluor 647 labelled anti CD45 antibodies and backfilled with either BSA, PLL-PEG or F127. On these substrates, denominated “direct micro-contact printing”, the time for the T cells to spread was rather large, usually more than 1 h and reproducibility of the spreading was not achieved, the substrates showing often incomplete patterns or even imperfect cellular adhesion as seen in RICM. We concluded that either we were losing a large amount of antibodies while patterning, resulting in the patterns being polluted by the repulsion molecules used for backfilling, or that our antibodies were sensitive to the drying step needed before stamping, hence being partially damaged by the process. Quantifying the fluorescence of anti CD45 antibodies stamped to a glass substrate, after incubation on a stamp, to the one of adsorbed antibodies, we estimated that the loss could be ~ 40–50% as compared to a simple 45 min adsorption procedure (Suppl. Fig. 2)^66^.

As a consequence, we decided to stamp repulsive molecules first with inverted patterns corresponding to the previous ones, then backfill them by adsorbing anti CD45 antibodies (Fig. 2c). Notably, we did not succeed in transferring efficiently PLL-PEG or pluronic F127, even by modifying either the stamp or the glass substrate using plasma activation or UV exposure. Aside, we obtained good results when the repulsive proteins were BSA or biotinylated BSA onto unmodified or plasma activated glass (Fig. 2d).

We then optimized the protocols to obtain the higher adhesive/repulsive contrast when cells were seeded onto bi-functional substrates. We patterned biotinylated BSA, adsorbed anti CD45 antibodies and finally functionalized the biotinylated BSA with fluorescent streptavidin (Fig. 2d). This solution afforded us to use unlabeled anti CD45 antibodies in conjunction with labeled streptavidin in order to reveal the proper (inverse) patterning of our substrates while avoiding any fluorescence cross-talk for recording calcium probe signals.

To be compatible with our planned mechanical tests using AFM, we wanted to (a) minimize the time needed for T cells to adhere on the larger stamps of anti CD45 antibodies, (b) adjust the temperature during spreading to minimize calcium dye compartmentalization and (c) wash gently the non-adherent cells away while avoiding displacing/perturbing the patterned ones. We determined that an incubation of the cells for 1 h on the patterned surfaces at 25 °C, when previously pre-loaded with the calcium reporter molecule, allowed a good compromise between criteria (a) and (b). Thus, the protocol presented in the Material and Methods section was the one we observed to be optimal and tractable for our experimental needs.

Cells were spreading circularly as expected on the patterns, exhibited similar areas on the largest patterns (2R ~ 20 µm) and on continuous substrates (314 µm^2^ vs. 322 µm^2^, resp.) but different cell morphologies : contact zones, were more elongated (shape index ~ 0.6 ± 0.2; see Suppl. Fig. 2 and Fig. 2a for continuous vs. Fig. 2d,e for patterns) and cells were flatter with lower apical membrane to nucleus distance (Fig. 2f) on continuous adherent substrates. They did not exhibit strong alterations of their behavior and liveliness: they appeared to have a dynamical membrane, but were not motile even on continuous substrates. All in all, they were properly immobilized and could be kept alive up to 3 h for non-labeled cells before starting to present vacuoles (not shown), a very interesting point for AFM measurements as described hereafter.

### T cell mechanics of patterned T cells

**Figure 3:**
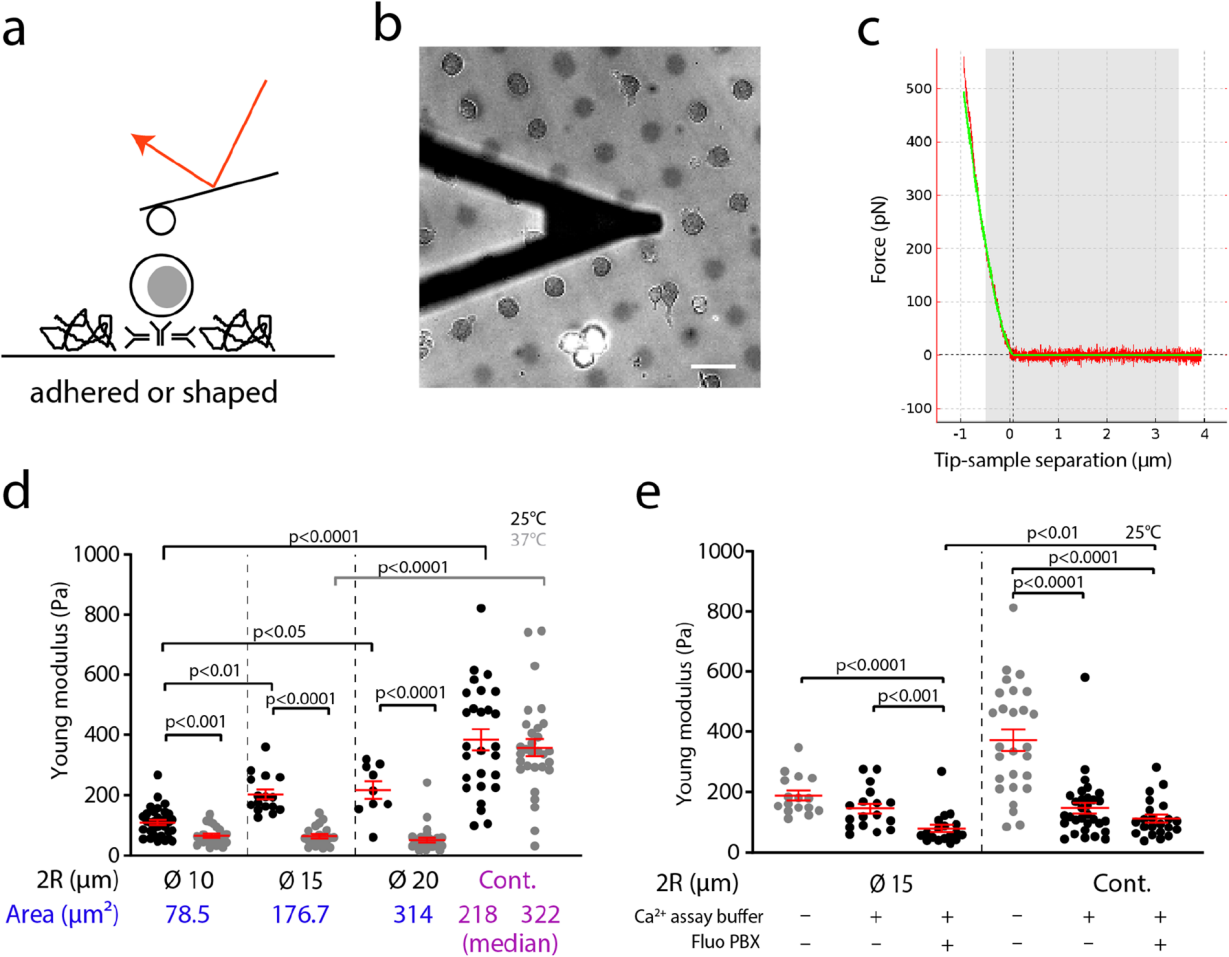
(**a**) Schematics of AFM indentation experiments on patterned 3A9m cells. (**b**) Micrograph (bar = 20 µm) of a bead decorated lever (black triangular shadow) close to patterned 3A9m cells. The image is an overlay of the fluorescence of the non adhesive zone and of the corresponding brightfield image. (**c**) Representative indentation force curve (only the pushing part), with a Hertz fit (green curve) for a spherical indenter over the first 0.5 µm of indentation (grey zone). (**d**) Young modulus measurements of patterned and non-patterned cells performed at 25 °C and 37 °C. (**e**) Effect of the incubation of the PBX dye and loading buffer on the measured Young modulus (at 25 °C) of the patterned and non-patterned cells. In grey, data from (**d**) for comparison. (**d**, **e**): one point is the average modulus obtained over up to 10 repeated indentations of the same cell. Red symbols are for mean ± SEM. Significance of difference is assessed with a Mann–Whitney test. Continuous substrate is denominated as “cont.”.

Cell mechanics was characterized by AFM indentation using bead decorated cantilevers (Fig. 3a–c). We present on the x-axis of Fig. 3d the typical adhesion areas that cells can develop. We repeatedly indented adhered cells with a moderate maximum contact force, with contact times of 0 s or in some cases up to several seconds. In the first case, we extracted the Young modulus from the contact part of the force curve^43,58,67^, and in the second case we also extracted the exponent of a power law fit of the force relaxation during the first seconds of contact, which relates to the cell viscous behavior^60^.

In order to set the maximal indentation depth for the Hertz fit, we evaluated using confocal microscopy z-stacks the relative positions of the apical membrane/cortex of actin and of the nucleus on adhered cells (Fig. 2e,f). We obtained typical values of 1 µm or more and consequently chose to fit the experimental indentation curves over the first 0.5 µm of indentation to exclude as much as possible any contribution due to the nucleus.

As mentioned previously, we used a step of adhesion and spreading at 25 °C over the decorated substrates in order to prevent the cells, when loaded with the calcium dye, to sequester it in intracellular organelles, precluding a robust use for calcium reporting. We then performed the mechanical experiments at 25 and 37 °C. Our measurements confirmed that temperature was a crucial parameter for T cell mechanics (Fig. 3d) as for other leukocytes^68^. We observed that, when indentation experiments were performed at 25 °C, the measured Young modulus gradually increased following the spreading area (Fig. 3d). In contrast, when the experiments were performed at the physiological temperature of 37 °C, the only marked difference was observed for patterned vs. continuous substrates, the three pattern sizes inducing no visible change or trend in Young modulus. The values we obtained for constrained cells at 25 °C or at 37 °C were coherent with the published values for T cells that were only weakly aspirated in micropipettes, without adhesion, suggesting that our method did not affect substantially T cell mechanics as long as activation was controlled^66^.

For the mechanical behavior of patterned cells, our results point out toward a possible mechanical homeostasis, being efficient at 37 °C, for the time during which the sample are prepared^69^. Interestingly, the difference between patterned and non-patterned cells could be linked to the morphology after spreading, since non patterned cells were flatter than patterned ones. Aside, the membrane/nucleus distance was observed to be significantly smaller for non patterned than for patterned cells (Fig. 2e,f). This may point toward a residual influence of the nucleus on the measured mechanics e.g. by differential densities or organization of (sub-membrane/above the nucleus) actin^70^, but our confocal imaging did not allow us to resolve such an organization.

To determine if our adhesion strategy was impacting other mechanical parameters of T cells, we performed force relaxation tests and quantified the characteristic power law exponent by an ad-hoc exponential decay fit to the force vs. time data segment^60^. This parameter was weakly sensitive to the modality of immobilization of the cells and the estimated values were, interestingly, on the same order of magnitude of the ones reported for other cell types^60^, resp. − 0.14, − 0.17, − 0.15, − 0.09 (median value) for stamps of 10, 15, 20 µm in diameter and continuous substrates at 25 °C. The ranking of these values may suggest that the patterned T cells exhibited a similar viscous behavior while the ones spread on continuous substrate could be considered as slightly more viscous.

Moreover, the loading of T cells with the calcium probe did not influence neither T cells shape nor adhesion. Surprisingly, their Young modulus was slightly lower than for non-loaded cells of the same shape and spreading. We identified this perturbation to be the effect of the commercial loading solution provided with the reporter dye which may contain some tensioactive molecules to help the dye permeate (Fig. 3e) and could not rule out that the slight calcium sequestration by the probe may on top introduce a perturbation of the cytoskeleton architecture^71^.

### Shaping T cells through anti CD45 patterns does not affect their activation by soluble anti CD3 antibodies

**Figure 4:**
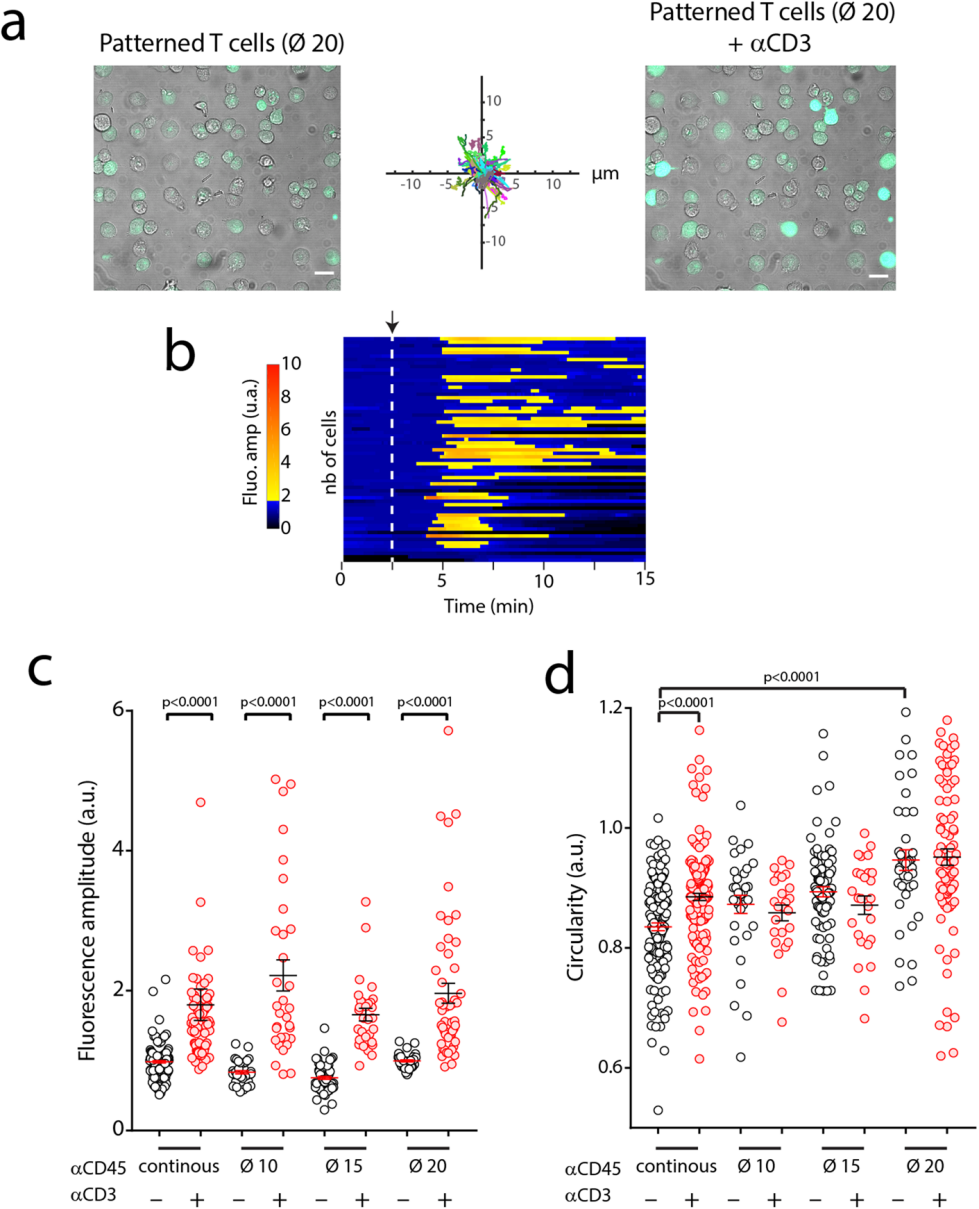
(**a**) MAAACS experiments with PBX loaded 3A9m cells sitting on 20 µm anti CD45 antibody patterns. Representative images before and after addition of soluble anti CD3 antibodies (scale bar = 20 µm). Re-aligned trajectories, showing that cells are essentially immobile. (**b**) Heatmap of fluorescence amplitude vs. time of only patterned cells, with the instant of anti CD3 antibody addition shown by a dashed line. One line is one detected cell. (**c**) Fluorescence amplitude before and after injection of anti CD3 antibodies for cells patterned or not. (**d**) Circularity variation with the introduction of anti CD3 antibodies, showing that non shape restricted cells are rounding upon activation with soluble anti CD3 antibodies. One point in one cell. Bars are for mean ± SEM. Significance of difference was assessed with a Mann–Whitney test.

Using MAAACS, we observed that soluble anti CD3 stimulation of patterned cells (i.e. after 1 hr spreading at 25 °C before returning the cells to 37 °C) induced robust calcium signals comparable to the classical MAAACS situation, where T cells were spread on continuous anti CD45 substrates (for the same duration) (Fig. 4).

Since the mechanical properties of the cells were similar at 37 °C among the patterns but not with the continuous situation, and that the largest patterns lead to a same spreading area than the continuous substrate, we propose that, in our conditions, calcium fluxes elicited by soluble stimulation were neither affected by cell geometry, cell elasticity nor cell anisotropy of spreading.

Interestingly, the amount of CD45 sequestered at the basal side of the cells does not appear to affect neither the calcium signal intensity nor the dynamics (i.e. the time delay before calcium rise or speed of this rise, as observed on heatmaps of fluorescence vs. time in Figs. 1b and 4b): from 10 to 20 µm in diameter for the patterns, the apparent adhesive area was increased by a factor 4, which could lead up to a possible 4-times larger consumption of CD45 molecules when estimated using a close packing distribution of antibodies on the surface and full binding.

### Ectopic expression of an non-immunologic adhesion molecule

**Figure 5:**
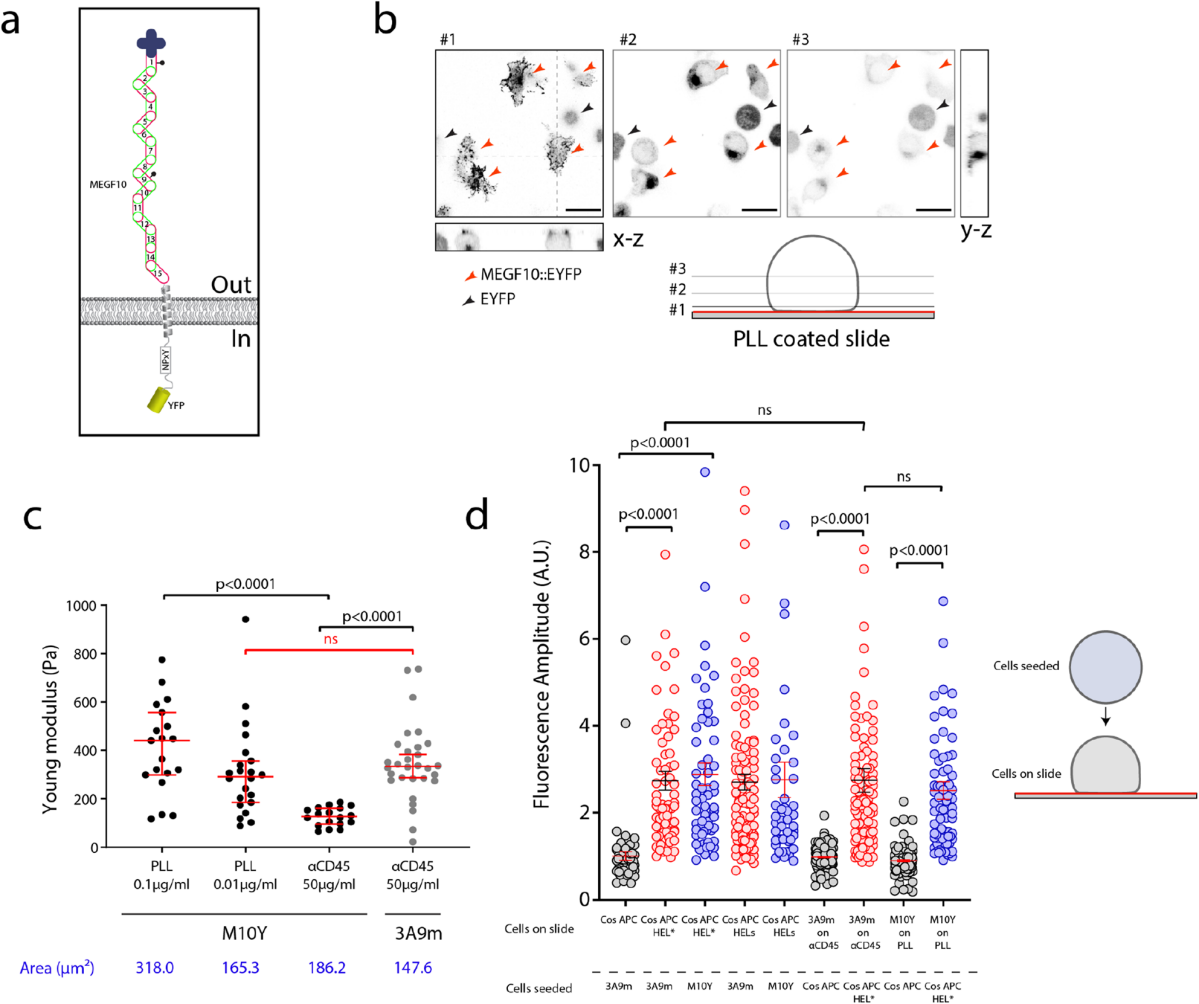
(**a**) Schematics of the structure of MEGF10 construct as a membrane receptor, labelled with YFP, expressed in 3A9m M10Y. (**b**) Confocal imaging of the polarised MEGF10 localisation when the cells are adhering on PLL substrate, showing that MEGF10 is mainly segregated at the contact zone(#1–3 confocal sections at various altitudes z); red arrows: M10Y expressing 3A9m T cells; black arrow: cytosolic YFP expressing 3A9m T cells (scale bar = 20 µm). (**c**) Variation of the adhesion area and mechanical properties of the transfected cells as compared to anti CD45 antibody continuous substrates case. For an intermediate concentration of PLL during the incubation, M10Y cells achieved to spread like WT cells on anti CD45 antibodies  and possessed the same mechanical properties. (**d**) Activation of M10Y vs. 3A9m cells when seeded on COS APC cells as in classical MAAACS experiments or when adhered respectively on PLL or anti CD45 and contacted by sedimenting COS APCs without antigen or I-Ak-HEL expressing COS-7. Results were expressed as mean ± SEM and statistical analysis was performed using a Mann–Whitney test.

To further verify that the impact of CD45 was minimal in our system, we ectopically expressed in 3A9m cells an adhesion molecule that is not expressed in T cells. We chose the MEGF10 protein (for Multiple Epidermal Growth Factor-like domain Protein 10, Fig. 5a)^49^. This protein is the orthologue of the Ced-1 receptor involved in the recognition of apoptotic cells in the nematode *C. Elegans*^72^. Various studies in Drosophila (DRAPER gene) or in higher eukaryotes have shown that this family of membrane proteins has retained this function^73^. However, it can be noted that the expression of MEGF10 in mammals is restricted to the cerebral (expressed in astrocytes^74^, in retinal cells^75^) and muscular spheres where MEGF10 would influence the proliferation of muscle cells^76^.

We electroporated the original MEGF10::YFP construction^49^ and selected a population of 3A9m cells expressing a comparable level of TCR as original 3A9m and a high level of YFP fluorescence (3A9m M10Y). We obtained a polyclonal population of mixed cells that express either MEGF10::YFP (M10Y+) or only cytosolic YFP (due to a truncated integration of the construction and a shift of an initiating codon at the ATG level of the YFP) (Fig. 5b). However, this M10Y + population demonstrated a very strong adhesion to poly-l-Lysine (while very weakly adherent on glass alone), and an enrichment of the MEGF10::YFP protein at the basal membrane in contact with the support, in contrast to cytosolic YFP expressing 3A9m T cells that loosely adhere without spreading on this substrate (consistent with observations for 3A9m T cells in a previous section). The M10Y + T cells spread very quickly (between 5 and 10 min) in the form of a dotted veil with local enrichments as spikes, and we noticed the additional presence of small filopod-type structures (Fig. 5b), consistent with the literature^77^. In comparison, cells expressing only cytosolic YFP did not show this enrichment or membrane extensions (Fig. 5b).

Varying the concentration of poly-l-Lysine during substrate preparation, we observed that a high concentration of PLL of 0.1% leads to a greater spread (almost a factor 2 on the area determined by RICM) compared to a concentration of 0.01% (Fig. 5c). In the former case, the cell was more rigid, as shown by a higher Young's modulus. On the other hand, the adhesion of the cells on a coated surface with a conventional concentration of poly-l-Lysine (0.01%) induced adhesion area (as measured by RICM) as well as Young modulus values of the same order as those measured for 3A9m spread on anti CD45 antibodies (Fig. 5c).

Interestingly when spread on continuous anti CD45 substrates, the adhesion area of M10Y + T cells appears to be close to the one of 3A9m cells, but these cells were softer than all other spreading conditions. One can only hypothesize that, when MEGF10 was not used for adhesion and seggregates to the contact zone, it may perturb the cytoskeleton organization and/or mechanical measurements due to its large extracellular domain.

### Immobilizing T cells strategies do not perturb their activation by APC

We investigated whether T cells made adherent onto functionalized surfaces would be still activated by antigen presenting cells. To that purpose, we used surrogate APC, namely COS 7 cells expressing the α chain of MHC II and the β chain of MHC II I-A^K^ alone (denoted COS APC) or covalently fused to the a peptide derived from the Hen Egg Lysozyme HEL (denoted COS APC HEL*)^42,48,50,78^. Alternatively COS APC were loaded with soluble antigenic peptide (aa 48–63) derived from HEL (denoted COS APC HEL).

We performed two symmetrical experiments. First, the 3A9m T cells M10Y + were adhered to the PLL coated surface, then a suspension of COS APCs was added and let fall by gravity on the spread T cells on continuous substrates. For comparison, 3A9m cells were seeded on surfaces covered with anti CD45 antibodies. Conditions were matched to have T cells spread similarly, hence having the same mechanical properties (see above). Second, conversely, COS APC cells and variants were adhered at the bottom of the observation chambers, and 3A9m or M10Y cells being seeded over them in a similar way as previously^42,50^. In both cases, the T cells have been pre-loaded with PBX calcium reporter, and experiments were performed at 37 °C.

The percentage of activated cells in the configuration where the T cells were adherent was generally lower than in the reverse situation (immobile APC and sedimenting / mobile T cells). This difference can be explained by the T/APC cell ratio since a good number of T cells present in the field of observation were not in contact by seeded COS APC cells. Monitoring the calcium fluxes, we observed that the fluorescence amplitude of M10Y + T cells (on PLL; FA = 1.96 ± 0.20) was highly similar to than the 3A9m adhered to on anti CD45 (FA = 1.73 ± 0.11; p = 0.498, Mann–Whitney test). Globally, whatever the configuration chosen or the experimental conditions, we could not find any significant difference between the activation signature of 3A9m cells and the M10Y + T cells by model APCs (Fig. 5d).

These observations suggested that at equivalent expression level of TCR, immobilizing CD45 on wild type 3A9m T cells did not impact significantly the antigen-dependant calcium release compared to M10Y + T cells.

In addition, we tried to obtain patterned surfaces with PLL as an adhesive molecule, by a direct printing strategy using modified stamps. It appears that in our hands, and with the glass bottom Petri dishes we used to be fully compatible with AFM measurements at 37 °C, the transfer of PLL onto the surface was not homogeneous (as seen using with a 9:1 mix of PLL:Alexa Fluor 546- labelled PLL), some part remaining on the PDMS stamp. We have tested the use of plasma treated the surfaces of glass petri dishes which slightly improved the quality and reproducibility of the transfer. We also tried to first incubate PDMS stamps with 10% Sodium Dodecyl Sulfate (SDS) before inking, since it was shown that such pretreatment may increase the proportion of transferred PLL onto an activated glass surface^79^, with, again, a moderate success.

Moreover, it unfortunately proved to be difficult to obtain functional patterns, suitable for the immobilization of M10Y cells since they also tend to adhere relatively strongly to bare glass and also, surprisingly, to BSA: we tested in particular PLL/PLL-PEG combinations, PLL-PEG being used as repulsive coating molecule to backfill the free space between the stamps (Suppl. Fig. 4). Incomplete patterns were observed without SDS pre-treatment of the PDMS stamp, which lead either to non-specific adhesion, with cells adhering everywhere, or to specific but rarely complete adhesion since, most probably, repulsive molecules were polluting the stamps. In the case of stamps pre-coated with SDS, the cells did not spread over the entire surface of the pattern, indicating again a potential contamination with the repulsive molecules.

These limitations, in parallel with our results on the mechanics and activability of the cells, has confirmed our idea that the H193.16.3 anti CD45 antibody is, in our hands, one of the simpler and better ways to shape murine T cells onto patterns.

## Conclusions and perspectives

Here we presented a strategy aiming at controlling T cell shape and mechanical properties, while maintaining a good activability. We used model murine hybridoma T cells which recapitulates the essential steps of T cell activation up to IL-2 production. Using a refined image processing method, named MAAACS, we identified several molecules which could be used to immobilize cells on a glass substrate, and several preventing this adhesion, of which the couple (anti CD45 antibody, BSA/biotin BSA) was chosen. We determined by interference microscopy recording the maximal spreading of the cells on continuous substrates.

We tested direct micro-contact printing of the anti CD45 antibody and observed that reproducibility of the adhesive behavior was non ensured, and attributed this to a potential loss or denaturation of the antibody during the necessary drying step of our procedure. We then designed an “inverse” printing protocol allowing us to first shape the repulsion, then adsorb the adhesive molecule, and reveal the repulsive areas using labeled streptavidin, avoiding any interference with calcium fluxes detection on the shaped cells. Patterning the repulsion and building the adhesive zones by adsorption also helped us ensure that the amount of anti CD45 is the same as on continuous substrates, the extension of the adhesive zones allowing us to control how many molecules we propose to the cell to adhere on to test the influence of anti CD45 immobilization on our activation assay.

We then characterized cell mechanics by AFM indentation with moderate forces and noted that spreading on circular patterns of anti CD45 does not influence cell elasticity at 37°C. In contrast, asymmetrical cells obtained on continuous substrates, with a comparable adhesion area as for the largest patterns, were more rigid, more likely due to a different tension, skeleton organization or positioning of the nucleus. We observed that temperature, but also preliminary incubation with the loading buffer of the calcium dye, has an effect on cell mechanics, with Young modulus being the lower when the solution was used and the cells measured at physiological temperature. The dye itself, even if chelating some of the cytosolic calcium, had only a mild effect on the elasticity.

When tested for activation behavior with anti CD3 in solution, patterned cells and non-patterned cells exhibited the same capabilities, indicating that in our assays CD45 consumption by adhesion was not having a strong impact. The key point was to allow the T cell spreading on patterns while maintaining a level of calcium dye relocalization from the cytosol as low as possible by a careful play on the temperature during the spreading phase.

To rule out that partial CD45 immobilization would affect T cell activation, we generated a new cell line, expressing a non immunological adhesion molecule that allowed strong adhesion to PLL substrates, compared to wild type 3A9m, even if PLL is not unequivocally considered as the ideal experimental substrate for lymphocyte adhesion^80^. MEGF10 was shown to localize at the contact between cells and substrates. By tuning the amount of PLL used to decorate the substrate, we found a condition where the spreading area together with the Young modulus of this new cell line were similar to the ones of the original cell line when spread on continuous anti CD45.

We verified that, when challenged by model APCs, the two cell lines immobilized on anti CD45 or PLL (resp.) were exhibiting the same activation capabilities, underlying the fact that in our experiments, the use of CD45 as an anchor to the surface does not affect activation patterns and intensity as measured using calcium fluxes reporters.

The “platform” of the calcium probe loaded and shaped T cell we designed, with controlled mechanical properties, opens up new possibilities for single molecule approaches such as Single Cell Force Spectroscopy for excluding shape and mechanical effects. It also permits more complex mechanical characterization of the impact of soluble molecules on cell membrane or cytoskeleton, such as by pulling tethers with optical tethers where it has been hypothesized that initial state of the cell influences measurements, and potentially opens up way to study T cell mechanics modulation by controlled surface activation signals (density, amount, shape). It also provides periodic, calibrated T cells for refined imaging such as PALM/STORM or diffusion measurements such as Fluorescence Correlation spectroscopy. By varying T cell shape using asymmetrical patterns, more migrating like shapes of cells could be obtained, allowing to study the impact of cell shape, and not only spreading, on cell function in a reliable manner.

## Supplementary Information

Supplementary information.
