# Supplementary material for: Controlling T cells spreading, mechanics and activation by micropatterning
Source: Sci Rep. 2021 Mar 24;11:6783. doi: 10.1038/s41598-021-86133-1 (PMC7991639; doi:10.1038/s41598-021-86133-1)

**Controlling T cells spreading, mechanics and activation by micropatterning**

Anaïs Sadoun ^1-4^, Martine Biarnes-Pelicot ^1-3^, Laura Ghesquiere-Dierickx ^1-3,#^, Ambroise Wu ^4,§^, Olivier Théodoly ^1-3^, Laurent Limozin ^1-3^, Yannick Hamon ^4,*^ , Pierre-Henri Puech ^1-3,*^

^1-3^ Adhesion and Inflammation Lab (LAI)

^1^ Aix Marseille University, LAI UM 61, Marseille, F-13288, France.

^2^ Inserm, UMR_S 1067, Marseille, F-13288, France.

^3^ CNRS, UMR 7333, Marseille, F-13288, France.

^4^ Centre d’Immunologie de Marseille Luminy (CIML)

^4^ Aix-Marseille University, CNRS, Inserm , CIML Marseille, F-13288, France.

# present address : Division of Medical Physics in Radiation Oncology, German Cancer Research Center (DKFZ), Heidelberg, Germany.

§ present address : Department of Biophysics, University of Wrocław, Poland.

^*^ corresponding authors : [hamon@ciml.univ-mrs.fr](mailto:hamon@ciml.univ-mrs.fr) ; [pierre-henri.puech@inserm.fr](mailto:pierre-henri.puech@inserm.fr)

**Supplementary figure captions**

**Suppl. Fig 1 :** Serial Z-stacks of 3A9 T cells (from bottom to top) spread onto anti CD45 (a) or PLL (b) prior to surface labelling with AF488 anti CD45 (10µg/ml). Scale bar=15µm. (c) Quantification of fluorescence ratio between basal mean membrane fluorescence intensity over total mean cell fluorescence (in percentage). Alternatively to (a and b), cells were labelled prior being seeded onto anti CD45 decorated surfaces. Results are expressed as a dot plot (each dot corresponds to an individual cell) and the median is presented.

**Suppl. Fig 2 :** Comparison of the transfer efficiency of direct vs. inverse microcontact printing techniques (MCP) on glass bottom Petri dishes with anti CD45 coupled to Atto 565, in regard to simple adsorption, as a function of the solution concentration. Duration of incubation between inverse MCP and adsorption were the same (see text). Exponential fits to the data were plotted as a guide for the eyes when more than three data points were available.

**Suppl. Fig 3 :** Snapshots images taken from a video acquisition of 3A9 T cells (loaded with calcium indicator, as shown as overlay) spreading onto a continuous anti CD45 coated surface. Scale bar=20µm.

**Suppl. Fig 4 :** Stamping 9:1 PLL:Alexa Fluor 546-labelled PLL using PDMS stamps, pre-treated or not with SDS. Backfilling was performed using PLL-PEG. When no SDS was used, the transfer of patterns was poorly resolved. When SDS was used and M10Y cells seeded , the cells failed to completely fill the patterns which were better resolved. Scale bar=20µm.

**Suppl. Fig 1**


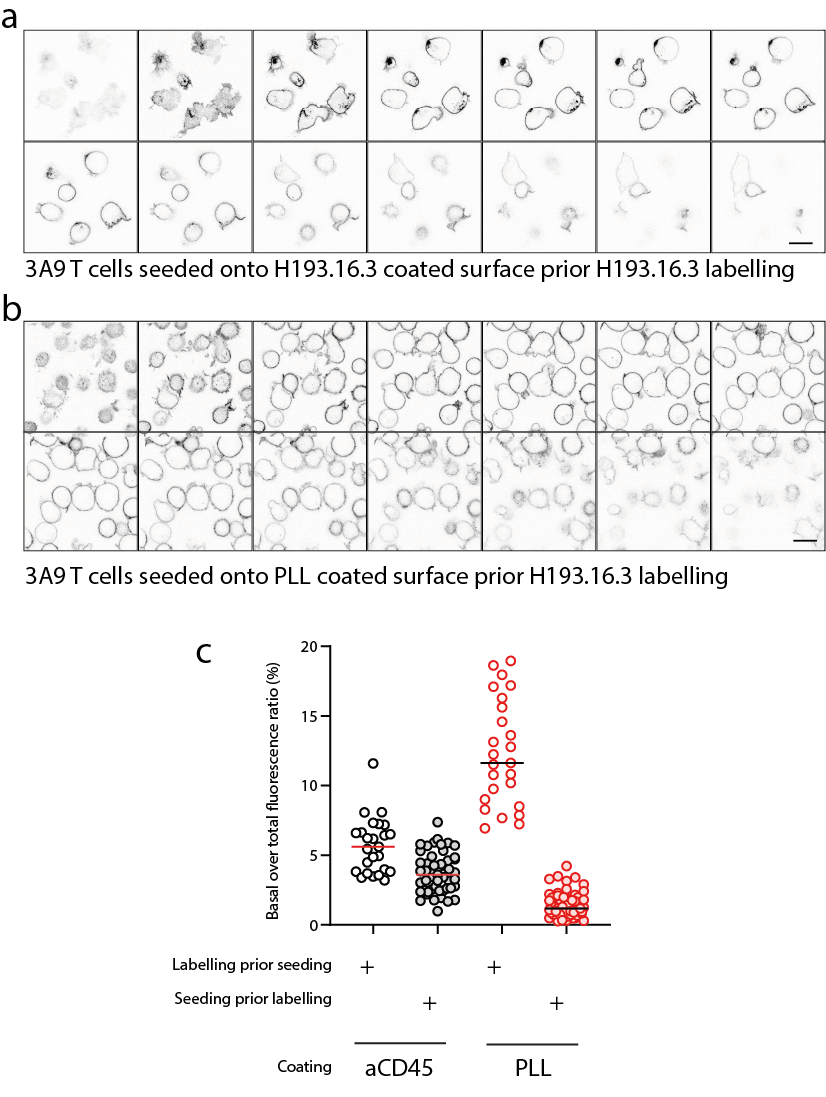


**Suppl. Fig 2**


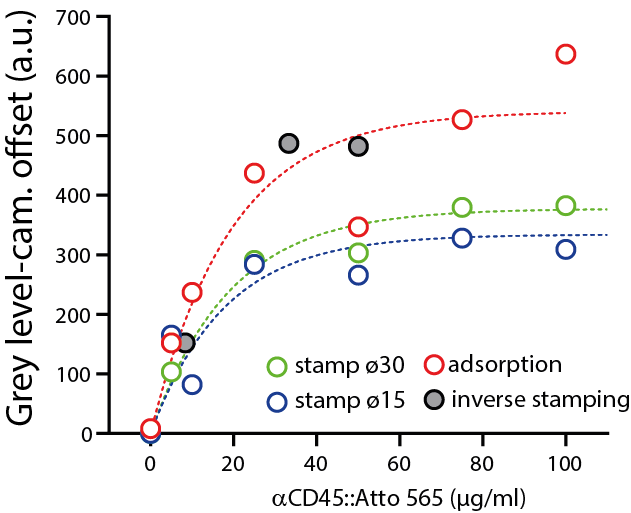


**Suppl. Fig 3**


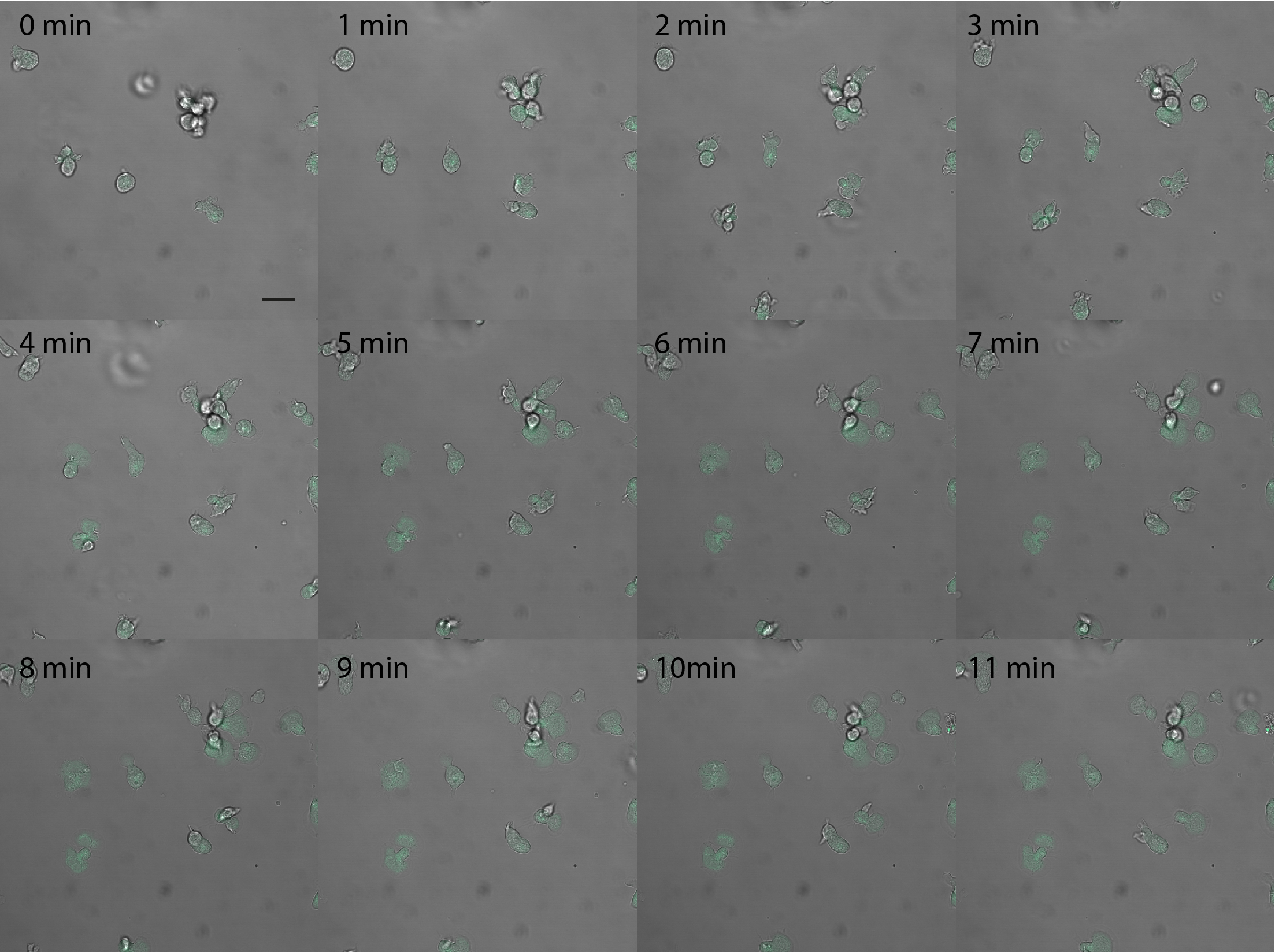


**Suppl. Fig 4**


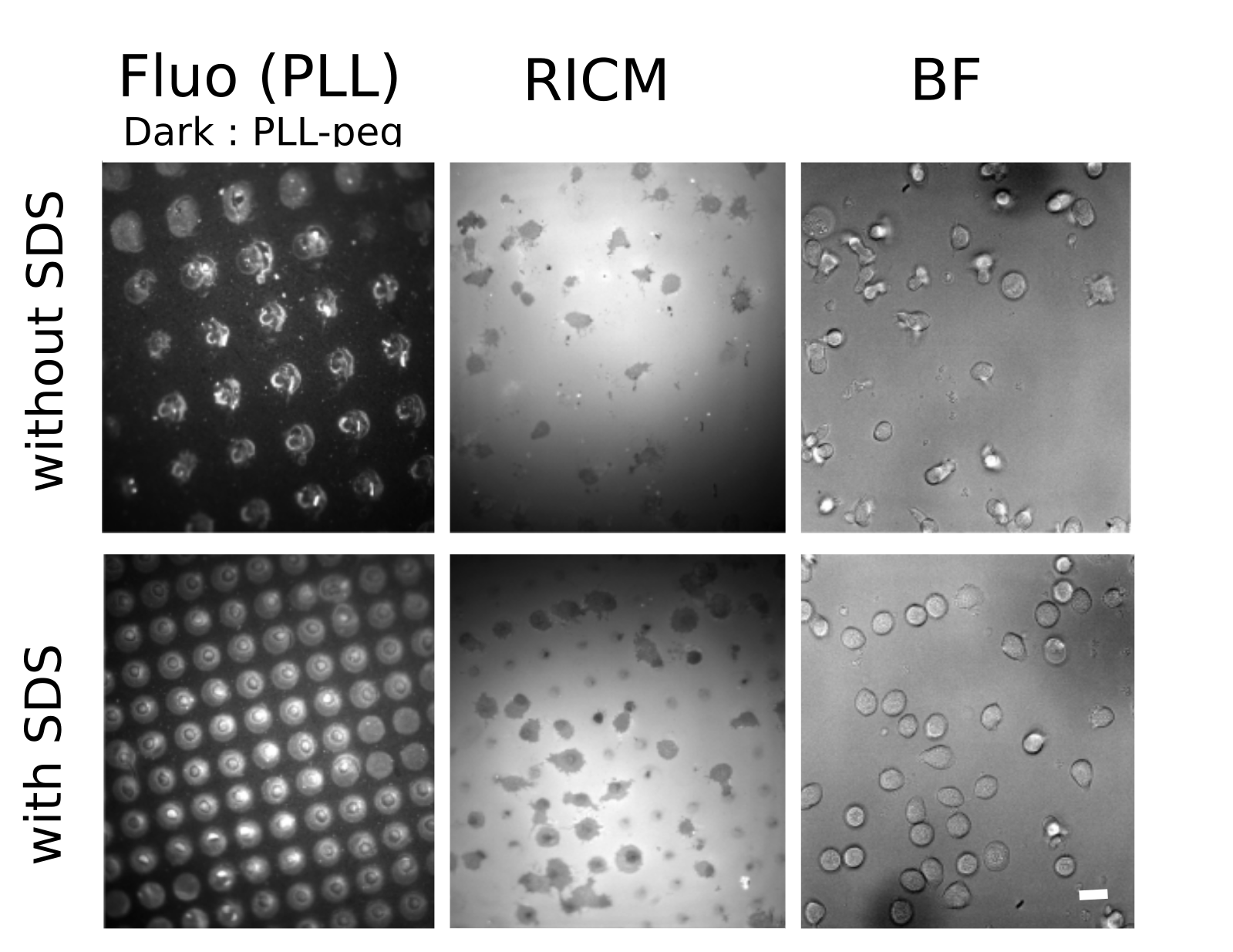

Supplement: Supplementary file 1 — Supplementary information. [file 41598_2021_86133_MOESM1_ESM.docx]
